# A novel apolipoprotein E mutation caused by a five amino acid deletion in a Chinese family with lipoprotein glomerulopathy: a case report

**DOI:** 10.1186/s13000-019-0820-6

**Published:** 2019-05-15

**Authors:** Weiji Xie, Yi Xie, Zhijun Lin, Xiaochang Xu, Yimin Zhang

**Affiliations:** 1 Department of Nephrology, The Second Affiliated Hospital of Shantou University Medical College, Shantou, Guangdong China; 2 Department of Nephrology, The Sixth Affiliated Hospital of Sun Yat-sen University, Guangzhou, Guangdong China

**Keywords:** Lipoprotein glomerulopathy, Apolipoprotein E, Mutation, Amino acid deletion, Kidney disease

## Abstract

**Background:**

Lipoprotein glomerulopathy (LPG) is a rare kidney disease with a poor prognosis that is related to mutation of the apoE gene. More than 10 variants of apoE associated with LPG have currently been identified.

**Case presentation:**

A male and his mother presented with proteinuria during a health examination. They went to hospital for further examination. Renal biopsy was performed, and the diagnosis was lipoprotein glomerulopathy (LPG), which is a rare, inherited renal disease. Medical histories were collected from the 2 LPG patients and their family members. The patients and family members underwent a routine urine test, and their renal function, blood lipids, and lipoprotein levels were examined. Genomic DNA was extracted from the peripheral blood of 7 family members, and exon 2, exon 3 and exon 4 of apoE were amplified by polymerase chain reaction (PCR). The purified PCR products were sequenced. Sequence analysis identified a 15 bp deletion (GCGCAAGCTGCGTAA) in exon 4 of the apoE gene that results in a novel 5 amino acid deletion in apoE (143 K-147R → 0). No mutations were found in exon 2 and exon 3 of the apoE gene.

**Conclusions:**

This family study suggests that a novel ApoE mutation (143 K-147R → 0) may be etiologically related to LPG, and other genetic or environmental factors may be associated with the occurrence of LPG.

## Background

Lipoprotein glomerulopathy (LPG) is a kidney disease associated with abnormal lipid metabolism. It was first reported by Saito et al [1] in 1989 and named according to its pathological characteristics. Proteinuria and edema are the most common clinical manifestations. LPG is characterized by markedly dilated glomerular capillaries that are filled with layered lipoprotein thrombi. Immunofluorescent examination showed these tissues to be apoB- and apoE-positive. Laboratory tests showed elevated levels of apoE. LPG is a rare disease, with fewer than 200 reported cases worldwide. The majority of patients with LPG are Asians, and the familial aggregation of LPG has been shown [2, 3]. The disease can eventually progress to end-stage renal disease, and the prognosis is poor. At present, the pathogenesis of this disease has not yet been fully elucidated, but the disease is related to the mutation of the apoE gene. To date, more than 10 variants of the apoE gene have been found in LPG patients. To further study the etiology of LPG, urinalysis, renal function, blood lipids level, lipoprotein levels, and gene sequencing were performed in 2 patients with LPG and their family members.

## Case presentation

**Fig. 1:**
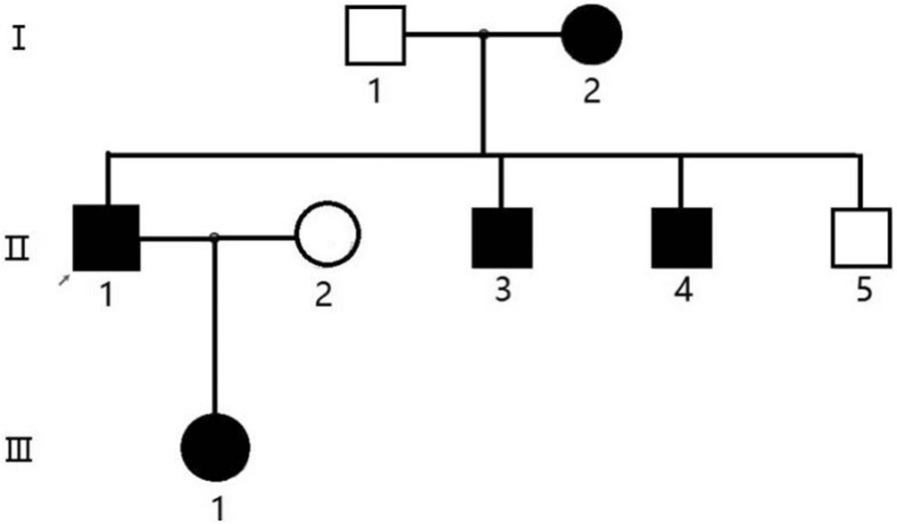
Family tree of the patient with apoE (143 K-147R → 0). Legend: The proband (II1) is indicated by the arrow. “ □ or ■ ” represent male,“○ or ●” represent female, blank symbols represent unaffected members, black symbols represent heterozygous for the apoE (143 K-147R → 0). I2 and II1 were pathologically confirmed LPG patients. II3 has nephrotic syndrome but did not undergo renal biopsy. II4 was diagnosed with IgA nephropathy. III1 is an asymptomatic carrier of the mutant

**Fig. 2:**
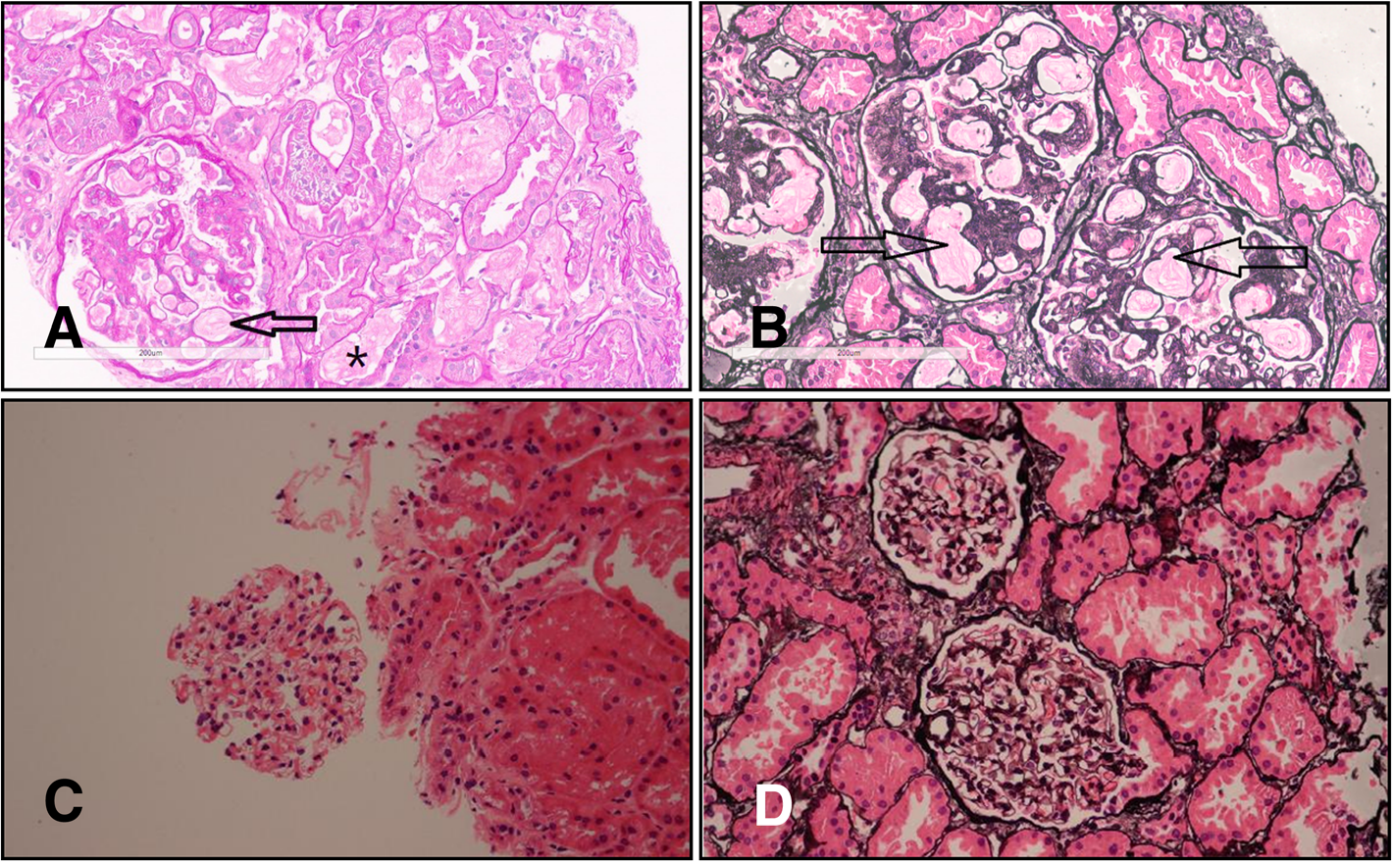
Light microscopy of glomerulus in the biopsy specimen of LPG patient II1 (**a**, **b**) and normal control (**c**, **d**). Legend: Glomeruli were significantly enlarged under light microscopy, glomerular capillary lumen was markedly enlarged and filled with pale-stained, thrombus-like material (**a**, **b** arrows)

**Fig. 3:**
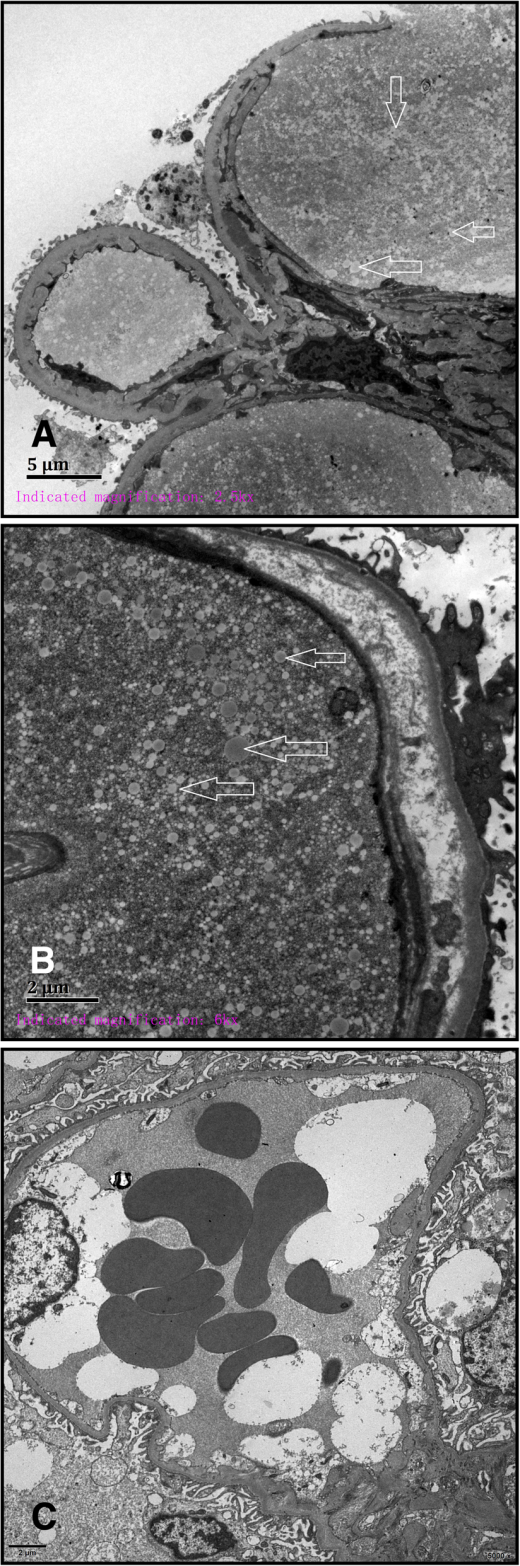
Electron microscopy of glomerulus in the biopsy specimen of LPG patient II1 and normal control (**c**). Legend: Under electron microscopy, glomerular capillary lumen was occupied with lipoprotein thrombi and there were a large number of lipid vacuoles in the thrombi (**a**, **b** arrows)

**Fig. 4:**
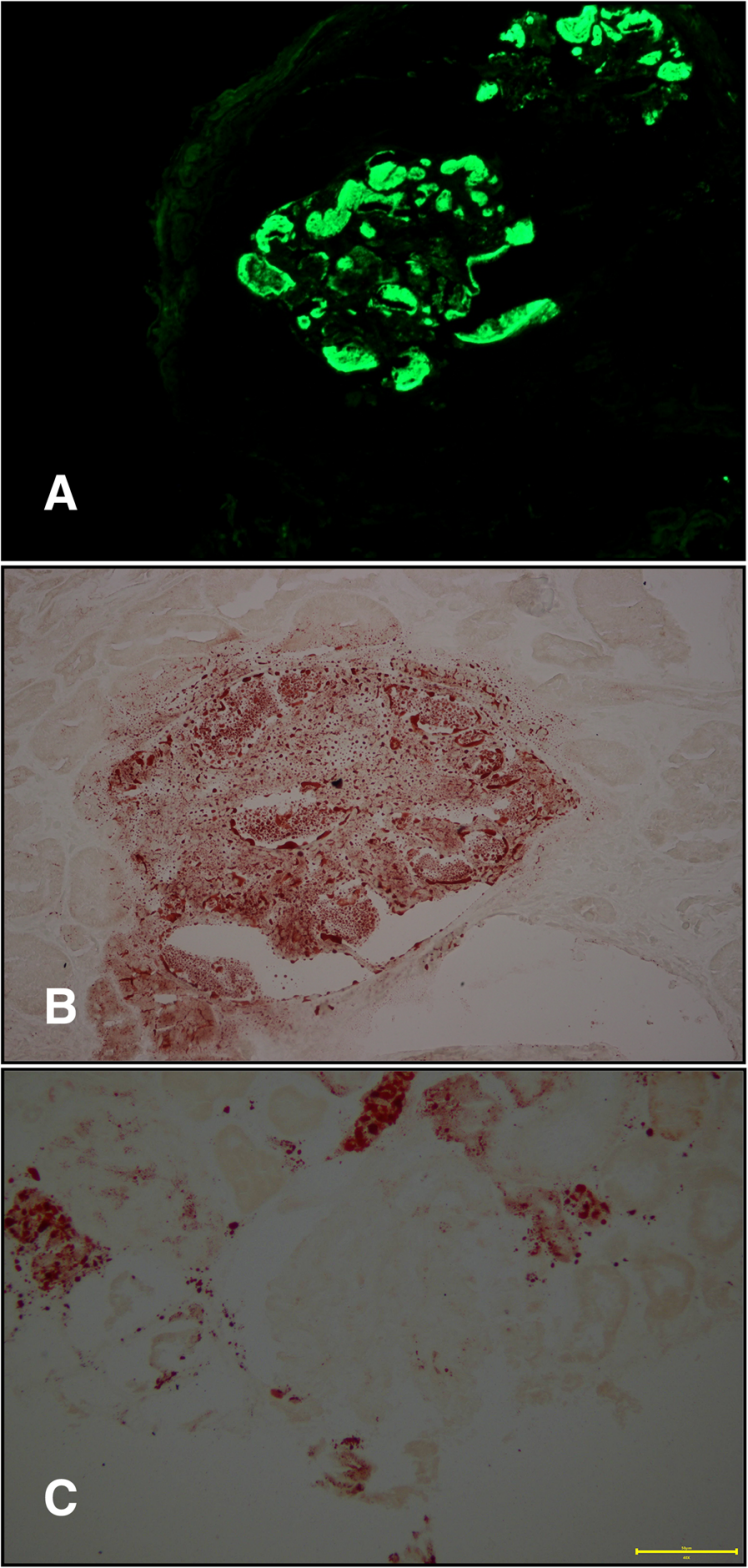
Immunofluorescent and Oil-red-O staining. Legend: Immunofluorescent showed stainings apoE are present in glomeruli (**a**). Oil-red-O staining showed lots of lipid droplets in glomerular capillary (**b**). **c** shows an oil-red-O staining glomerular capillary of the normal control specimen

We enrolled a family with 8 members in total, 2 of which were diagnosed with LPG. The proband’s wife (Fig. 1, II2) was not involved in this study (Fig. 1). The proband, a male (Fig. 1, II1), presented with proteinuria during a health examination approximately 4 years ago. He was referred to hospital for further examination. Urinalysis showed a qualitative urine protein level of 3+ and an occult blood level of 3+. Blood examination revealed 28.9 g/L albumin and 88 μmol/L creatinine. A renal biopsy was performed in order to further clarify the cause of the abnormal results, and the pathological diagnosis was LPG. At the time of his visit, the patient was treated with fenofibrate. The proband’s mother (Fig. 1, I2) first presented to the hospital with a 9 year history of edema and a 4 year history of hypertension. Laboratory investigations revealed a qualitative urine protein level of 2+, 31.85 g/L albumin, and 85 μmol/L creatinine. A renal biopsy was performed, and the diagnosis was LPG. The pathological findings of the two patients were as follows: the glomeruli were significantly enlarged under light microscopy, and the glomerular capillary lumen was markedly enlarged and filled with a pale, thrombus-like material (Fig. 2 a, b arrow). Oil red O staining showed many lipid droplets in the glomerular capillaries (Fig. 4b). A small number of renal tubular lumen were occupied by a lightly stained, vacuolar thrombus-like material (Fig. 2a, asterisk). Under electron microscopy, the glomerular capillary lumina were occupied with lipoprotein thrombi, and there were a large number of lipid vacuoles in the thrombi (Fig. 3 a, b). Immunofluorescence showed strong staining for apoB and ApoE. (Fig. 4 a).

The proband’s mother had two sisters and two older brothers diagnosed with uremia, of which the eldest brother had died, and their specific medical histories were unknown. The proband’s younger brother (Fig. 1, II3) exhibited clinical manifestations of nephrotic syndrome, and a renal biopsy was not performed at the time. The proband’s second younger brother (Fig. 1, II4,) was diagnosed with IgA nephropathy (Lee grade V, Oxford classification: M1E0S1T2) 3 years ago. The proband’s father (Fig. 1, I1) had no clinical manifestations, and laboratory investigations revealed hyperlipidemia and urinalysis. The proband’s youngest brother (Fig. 1, II5) and the proband’s daughter (Fig. 1, III1) also showed no signs of nephrotic syndrome.

To further study genetic mutations in the family, we sequenced exons of the APOE gene in its members (except for the proband’s wife, Fig. 1, II2). The specific experimental steps were as follows. Genomic DNA was isolated from the family members’ peripheral blood using the TIANamp Blood DNA Kit (Tiangen Biotech Co., Ltd., Beijing, China). Ten microliters of the extract was used as a template for polymerase chain reaction. A reaction mixture 50 μL in volume with 0.2 mM each dATP, dGTP, dCTP, dTTP; 1× PCR buffer for KOD-Plus-NEO; 1.5 mM MgSO4, and 1 U KOD-Plus-Neo (Toyobo Co., Ltd., Osaka, Japan) was used for PCR amplification. OLIGO software was used for primer design (Table 1). The reaction conditions were as follows: 95 °C initially for 5 min, followed by a denaturation step at 95 °C for 30 s, annealing at 62 °C for 30 s and polymerization at 72 °C for 90 s for 35 cycles. The products were subjected to 1.5% agarose gel electrophoresis, and then the DNA was purified by a Tiangen Universal DNA Purification Kit (Tiangen Biotech Co., Ltd., Beijing, China). The purified PCR products were sequenced using an ABI 3730 automated DNA sequencer.

**Table 1:** Primers for exon 2, exon 3 and exon 4 of apoE gene

Primers		Primer length	Fragment length
APOE-EXON2-F	TGGTAAATGTGCTGGGATTAGGCT	24	362
APOE-EXON2-R	AGGCCCAGAGAGCGTCAAAT	20	
APOE-EXON3-F	CACCATGGCTCCAAAGAAGCATTT	24	395
APOE-EXON3-R	GACTTAGCGACAGGGGCAGAA	21	
APOE-EXON4-F	GCCTCCTAGCTCCTTCTTCGTC	22	1128
APOE-EXON4-R	CAGTATGTGGGCAGAAAGAGAAACT	25	

**Fig. 5:**
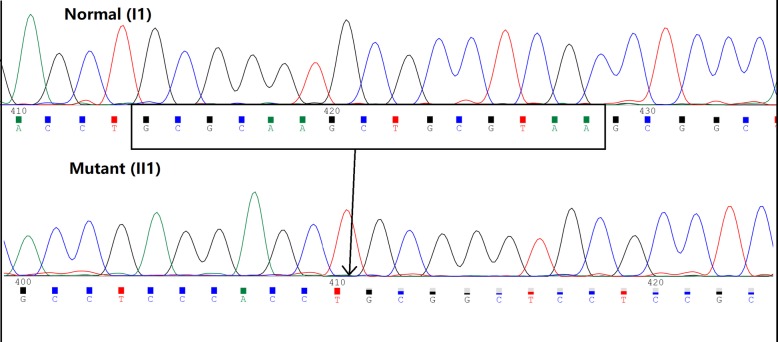
Nucleotide sequence of a segment of exon 4 in the apoE gene from both alleles of the proband (II1). Legend: The upper panel shows the normal apoE sequence. The 15 bp deletion apoE sequence is shown on lower panel

Gene sequencing shown a 15 bp deletion (GCGCAAGCTGCGTAA) in exon 4 of the apoE gene in the proband resulting in a 5 amino acid deletion in apoE (143 K-147R → 0) (Fig. 5). The proband’s mother (Fig. 1, I2), younger brothers (Fig. 1, II3–4), and daughter (Fig. 1, III1) also carried the same apoE mutation. However, no mutations were detected in the proband’s father (Fig. 1, I1) or his youngest brother (Fig. 1, II5).

To further gain clinical information about the family members, the subjects were instructed to fast, and blood samples were taken to test serum creatinine, serum albumin, total cholesterol, triglyceride, HDL-C, LDL-C, VLDL, apoE, apoA and apoB levels. Urine samples were taken for urinalysis. Increased urinary protein/creatinine ratios, proteinuria, serum lipids, and apoE levels were observed in proband and the proband’s mother, who were diagnosed with LPG. The proband’s younger brother (Fig. 1, II3) and second younger brother (Fig. 1, II4), who were carriers of the newly discovered apoE mutation, exhibited elevated TG, TC and LDL-C levels; proteinuria; and renal dysfunction. Moreover, the level of apoE in proband’s younger brother (Fig. 1, II3) was significantly increased. The proband’s youngest brother (normal apoE carrier, Fig. 1, I5) showed no abnormalities upon examination. The proband’s father (normal apoE carrier, Fig. 1, I1) had normal kidney function but elevated blood lipid and apoE levels. There were no abnormalities in the urinalysis, including serum creatinine levels, albumin levels, and blood lipid levels, of the proband’s daughter (mutated apoE carrier, Fig. 1, III1). However, unfortunately, for some reason, the apoE, apoA and apoB levels were not tested in the proband’s daughter. The results of detailed laboratory examinations of the family members are shown in Table 2.

**Table 2:** Laboratory examination results in apoE(143 K-147R → 0) family

	Gender	PCR	PRS	Cr	Alb	TC	TG	HDL-C	LDL-C	VLDL-C	apoE	apoA	apoB
		mg/mmol		umol/l	g/l	mmol/l	mol/l	mmol/l	mmol/l	mmol/l	mg/L	g/L	g/L
Carrier													
II1	Male	143.03	3+	104.42	39.4	4.76	2.18	1.36	3.04	1.04	73.9	1.28	1.16
II3	Male	654.62	3+	146.02	21.09	9.6	4.06	1.37	6.4	0.9	91.6	1.36	1.78
II4	Male	285.09	3+	190.35	39.33	5.63	2.84	1.61	3.39	0.18	48.6	1.33	0.62
II5	Male	7.53	–	77.02	47.89	5.73	1.4	1.32	3.85	0.48	39.1	1.22	0.91
Non-carrier													
I1	Male	7.21	–	112.03	42.94	5.54	2.39	1.04	3.63	1.51	72.9	1.35	1.23
I2	Female	551.77	3+	60.43	36.75	4.71	1.4	1.66	2.78	0.31	62.3	1.53	0.9
III1	Female	16.77	–	33.31	43.24	2.73	1.52	1.67	1.05	^a^	^a^	^a^	^a^
Normal range		<15		44–133	35–55	2.9–5.68	0.23–1.81	0.78–2.2	0.1–3.36	0.3–1.35	27–49	1.05–1.75	0.6–1.4

*PCR* Urine protein-to-creatinine ratio, *PRS* protein reagent strip results, *Cr* serum creatitine, *Alb* albumin, *TC* total cholesterol, *TG* triglycerides, *HDL-C* high-density lipoprotein cholesterol, *LDL-C* low-density lipoprotein cholesterol, *VLDL-C* very-low-density lipoprotein cholesterol. ^a^: For special reasons, the patient did not undergo the examination

## Discussion

ApoE, an alkaline single-chain polypeptide glycoprotein of 299 amino acids, binds to cell surface receptors in the LDL receptor family and participates in lipid transport and metabolism [4]. In humans, ApoE has three common isoforms, named apoE2, apoE3, apoE4, that are controlled by three alleles of epsilon 2, epsilon 3, and epsilon 4, respectively [5]. ApoE3 is one of the most common isoforms. The amino acid residues in apoE at positions 112 and 158 determine the isoform type. ApoE3 has Cys and Arg residues at positions 112 and 158, respectively. ApoE2 has Cys residues at positions 112 and 158, while apoE4 has Arg residues at both positions. ApoE3 and ApoE4 bind normally to LDL receptors, whereas ApoE2 exhibits binding defects that affect lipid metabolism [6].

In 1997, Oikawa et al [7] sequenced the apoE gene and found a mutation in the apoE gene that resulted in the substitution of arginine at position 145 with proline, which was named the apoE-Sendai mutation (Arg145Pro). This gene mutation is associated with the incidence of LPG. Ishimura et al [8] confirmed this observation by viral transduction of the apoE-Sendai gene into apoE-deficient mice, which produced the same renal pathological changes as those seen in human LPG. Since then, researchers have discovered more than 10 new apoE mutations associated with LPG. Most of them are point mutations including apoE-Sendai (Arg145Pro), apoE Kyoto (Arg25Cys) [9], apoE Tsukuba (Arg114Cys) [10], apoE Chicago (Arg147Pro) [11], apoE Okayama (Arg150Gly) [4], apoE Modena (Arg150Cys) [12], apoE Guangzhou (Arg150Pro) [13], apoE Las Vegas (Ala152Asp) [6], apoE Osaka/Kurashiki (Arg158Pro) [14, 15], and apoE Hong Kong (Asp230Tyr) [16]. Deletion mutations such as apoE Tokyo (Leu141-Lys143 → 0) [17] and apoE Maebashi (Arg142-Leu144 → 0) [18] are not commonly seen. Most ApoE deletion mutations are found in patients in China and Japan, and patients with these mutations are rarely seen in the Americas and Europe. Among the deletion mutations, the apoE-Sendai and apoE-Kyoto mutations are the most commonly reported. In our study, we discovered a novel variant of apoE in two lineal relatives with LPG (Fig. 1; II1, I2) and their family members (Fig. 1; II3, II4, III1). The 15 bp deletion is in exon 4 of the apoE gene and results in a 5 amino acid deletion of apoE at positions 143~147 (KLRKR). It is noteworthy that the position of the amino acid deletion in this novel apoE variant is close to those in apoE Tokyo (Leu141-Lys143 → 0) and apoE Maebashi (Arg142-Leu144 → 0) reported in Japan. Some Chinese LPG patients also contained the apoE Tokyo and apoE (Lys143-Arg145 → 0) mutations. Thus, the apoE amino acid sequence 143 to 147 locus is a common apoE mutation site in patients with LPG.

Most of the mutation sites associated with LPG are located in the apoE amino acid sequence between positions 141 to 158. In addition, the amino acids at positions 136 to 160 in apoE are involved in binding of apoE to the LDL receptor [19]. Therefore, the binding capacity of the apolipoprotein for the LDL-R is likely affected, resulting in the aggregation and deposition of lipoproteins in the glomeruli. In addition, changes in amino acid residues may significantly change the three-dimensional conformation of the protein, thus making it easier for lipoprotein molecules to form polymer structures [5]. Hoffmann M et al [20] found that the ability of apoE2 to bind to the LDL receptor and HSPG were significantly decreased, and the binding capacity of apoE Sendai was similar to that of apoE2. However, the ability of apoE Sendai to bind to cell surface heparin sulfate proteoglycans was not affected by its mutation. This may make it easier for apoE Sendai to accumulate in the glomeruli and form lipoprotein deposits. Some mutations, such as apoE Kyoto (R25C), are located outside the LDL receptor binding region, and the binding capacity of apoE to the lipoprotein receptor is only 10% of that seen with wild-type apoE [9]. The possible reason for this is that the twenty-five Arg residues exposed to the surface in apoE form disulfide bonds with each other, which results in the aggregation of apoE [21]. In addition, oxidative stress may also be involved in the pathogenesis of LPG. The receptor binding domain of apoE is related to the antioxidant activity of wild-type apoE [22]. Abnormally structured apoE may lead to the oxidation of apoE. Oxidized apoE is more likely to form aggregates.

In this LPG pedigree, the proband (Fig. 1, II1) and his mother (Fig. 1, I2), who had been diagnosed with LPG, had elevated levels of proteinuria and blood lipids, and their levels of apoE and apoB were also elevated to varying degrees. By gene sequencing, we found a novel variant of apoE in these LPG patients but did not detect the mutation in the proband’s youngest brother (Fig. 1, II5). Unsurprisingly, the proband’s youngest brother (Fig. 1, II5) showed no clinical nephrotic syndrome with no abnormalities based on laboratory examination, suggesting that the apoE gene mutation is associated with the pathogenesis of LPG, dyslipidemia and lipoprotein metabolism. The proband’s younger brother (Fig. 1, II3) had nephrotic syndrome, and laboratory examination showed proteinuria, hypoalbuminemia, hyperlipidemia and a high level of apoB; the same apoE gene mutation was also detected, so we cannot exclude that the patient may be diagnosed with LPG. Renal biopsy is required to confirm the diagnosis. It is worth noting that the same gene mutation was also detected in the proband’s daughter. However, this patient did not show clinical symptoms and had normal urine tests and blood lipid levels. This phenomenon is consistent with the previous literature, in which a similar finding was reported [8, 13, 23]. Currently, it is hard to predict whether she will show clinical manifestations of LPG in the future. Continued regular follow-up is needed. Interestingly, the same apoE gene mutation was also detected in the proband’s second younger brother (Fig. 1, II4). The patient clinically manifested nephrotic syndrome, but was diagnosed with IgA nephropathy by renal biopsy. In previous literature, cases of lipoprotein nephropathy associated with IgA nephropathy, membranous nephropathy, and lupus nephritis have been reported [24]. Therefore, we cannot rule out the possibility that the patient has IgA nephropathy and misdiagnosed mild lesions from lipoprotein nephropathy. Repeated renal biopsy is required to further evaluate whether the patient also has mild lesions from LPG. Previous studies from our group and others have shown that healthy carriers of apoE variants are very common, implicating that LPG is a dominant genetic disease with incomplete penetrance [5, 25]. The finding that FcRg-deficient mice has renal lesions that similar to those of patients with LPG suggests that FcRγ deficiency results in impaired macrophage function, eventually leading the development of LPG [26]. Thus, the pathogenesis of lipoprotein nephropathy involves multiple factors, among which apoE gene mutation is a major factor in disease development.

## Conclusions

A 15 bp (GCGCAAGCTGCGTAA) deletion in exon 4 of the apoE gene was discovered in 5 family members (the proband’s mother; the proband; the proband’s younger brother, second brother, and third brother; and the proband’s daughter), resulting in a 5 amino acid deletion in ApoE (143 K-147R), that has not been reported in previous literature and databases. This mutation may be the molecular basis for the pathogenesis of lipoprotein glomerulopathy in this family.

## Abbreviations

A: Adenine
Ala(A): Alanine
ApoE: Apolipoprotein E
Arg(R): Arginine
Asp(D): Asparticacid
C: Cytosine
Cys(C): Cysteine
Dntp: Deoxy-ribonucleoside triphosphate
G: Guanine
Gly(G): Glicine
HDL-C: High-density lipoprotein cholesterol
HSPG: Heparan Sulfate Proteoglycans
LDL-C: Low-density lipoprotein cholesterol
LDL-R: Low-density lipoprotein Receptor
Leu(L): Leucine
LPG: Lipoprotein glomerulopathy
PCR: Polymerase chain reaction
Pro(P): Proline
T: Thymine
TC: Total cholesterol
TG: Triglyceride
Tyr(Y): Tyrosine
VLDL-C: Very- low-density lipoproteins
